# Over 500 000 people on kidney replacement therapy in the European Union

**DOI:** 10.1093/ndt/gfag017

**Published:** 2026-02-06

**Authors:** Alberto Ortiz, Anneke Kramer, Vianda S Stel

**Affiliations:** Department of Nephrology and Hypertension, Health Research Institute-Fundación Jiménez Díaz University Hospital, Universidad Autónoma de Madrid (IIS-FJD, UAM), Madrid, Spain; RICORS2040-renal; Madrid, Spain; Departamento de Medicina, Facultad de Medicina, Universidad Autónoma de Madrid, Madrid, Spain; ERA Registry, Department of Medical Informatics, Amsterdam UMC location University of Amsterdam, Amsterdam, The Netherlands; Amsterdam Public Health Research Institute, Quality of Care, Amsterdam, The Netherlands; ERA Registry, Department of Medical Informatics, Amsterdam UMC location University of Amsterdam, Amsterdam, The Netherlands; Amsterdam Public Health Research Institute, Quality of Care, Amsterdam, The Netherlands

**Keywords:** chronic kidney disease, dialysis, epidemiology, kidney replacement therapy, transplantation

## Abstract

The burden of chronic kidney disease (CKD) is driven by mortality and the necessity of kidney replacement therapy (KRT). CKD causes one death every 20 seconds globally and is among the fastest growing causes of death. It is forecast to become the fifth most common global cause of death and the third in western Europe by 2050. It is estimated that 511 549 European Union (EU) inhabitants depend on KRT, with nearly two-thirds (≈310 000) treated by dialysis, and the remainder being kidney transplant recipients (≈200 000). KRT is provided by every EU country. France (96 317), Germany (77 900), Spain (67 604), and Italy (62 523) account for 60% of people on KRT in the EU. Prevalence per million population ranged from 560 (Luxembourg) to 2022 (Portugal). Germany (55 129) leads in the number of people on dialysis, followed by France (52 817), Italy (44 382), and Spain (29 879). In addition to differences in the relative number of dialysis and transplant patients, there were also EU-wide differences in modality of dialysis. The hemodialysis/peritoneal dialysis prevalence ratio ranged from 52.0 (Slovakia) to 3.4 (Sweden). The EU should be aware of the burden of KRT when designing regulations, such as the Accelerating Clinical Trials in the EU (ACT EU) and the EU Medical Device Regulation (MDR) or making decisions regarding the prioritization of diseases and the budget for research and healthcare.

Chronic kidney disease (CKD) is diagnosed when there is evidence of kidney injury (such as a urinary albumin-creatinine ratio >30 mg/g) or decreased kidney function (i.e. glomerular filtration rate, GFR <60 ml/min/1.73 m^2^) for longer than 3 months with an impact on health [1, 2]. CKD is one of the fastest growing causes of death. It is forecasted to become the fifth most common global cause of death and the third most common in western Europe by 2050 [3, 4]. Globally, CKD caused one death every 20 seconds in 2023 [5]. Any degree of CKD, even the earliest stages where kidney function remains normal, is associated with an increased risk of premature death and of multiple types of non-lethal cardiovascular events [1, 6]. The hazard ratio for premature death or cardiovascular events is 1.5 to 14 in patients with CKD not yet having kidney failure, aged under 65 years. These risks increase with decreasing eGFR and/or increasing albuminuria, as does the risk of progression to kidney failure requiring kidney replacement therapy (KRT): either dialysis or kidney transplantation. However, the hazard ratio of kidney failure is already 5.4 before either GFR or albuminuria values reach the threshold required to diagnose CKD, and increases up to 1457 in more advanced CKD stages [6]. This has led to calls for primary prevention of CKD aimed at maintaining kidney health [7]. For this purpose, the European Renal Association (ERA) has developed a holistic ABCDE framework for cardiovascular-kidney-metabolic (CKM) health assessment (Table 1) [8]. Maintenance of kidney health is conceptually similar to the well-established guideline-recommended primary prevention of other CKM diseases such as diabetes, atherosclerotic cardiovascular disease or heart failure [9–11].

**Table 1:** The ERA has developed a holistic ABCDE framework for CKM health assessment.

A. Albuminuria	An actionable diagnostic criterion for CKD. A urinary albumin-creatinine ratio value persistently >30 mg/g diagnoses CKD even if kidney function is normal. Based on albuminuria values, guidelines recommend the prescription of up to 5 kidney and cardiovascular protective drugs that have been shown to decrease kidney and cardiovascular risk.
B. Blood pressure	High values diagnose hypertension, an actionable condition. Treating hypertension improves kidney and cardiovascular outcomes.
C. Cholesterol	High cholesterol values are found in familial hypercholesteremia and high LDL cholesterol values are part of cardiovascular risk scores used in otherwise healthy people for risk stratification and therapeutic decision-making. Treatment of familial hypercholesteremia and high cardiovascular risk scores improve cardiovascular outcomes.
D. Diabetes	This means assessment of serum glucose. High serum glucose values diagnose prediabetes and diabetes. Treatment of prediabetes prevents diabetes while treatment of diabetes improves kidney and cardiovascular outcomes.
E. eGFR	An actionable diagnostic criterion for CKD. An eGFR value persistently below 60 ml/min/1.73 m^2^ diagnoses CKD even if kidney function is normal. Based on eGFR values, guidelines recommend the prescription of up to 5 kidney and cardiovascular protective drugs that have been shown to decrease kidney and cardiovascular risk.

This framework reproduces the items that healthcare providers should assess to implement the 2021 European Society of Cardiology Guidelines for cardiovascular disease prevention.

The ERA Registry has reported the epidemiology of KRT in Europe for >60 years [12, 13]. More recently, it has started comparing the epidemiology of KRT in Europe and the USA [14]. However, it has not yet produced data on the epidemiology of KRT in the European Union (EU). This is due in part to a lack of contributions from EU countries such as Germany, Bulgaria, Ireland, Slovenia, Slovakia, Malta, Luxembourg, and Cyprus, and incomplete datasets (e.g. missing kidney transplantation data) from other countries [13]. The lack of EU data represents a clear unmet need, given the supranational nature of the EU and its role in budget and legislative decisions that have direct impact on kidney health, research, and care. If the EU is blinded to the burden of CKD and, more specifically to the burden of kidney failure requiring KRT, an evidence-based, rational decision-making process may be compromised. The unmet need became clear as the EUDIAL working group of the ERA drafted a response to the European Commission call for evidence to revise the EU Medical Devices Regulation (MDR, 2017/745) [15–17].

The present report has estimated the prevalence of KRT in the EU in 2023 based on two sources: the ERA Registry in-house, publicly available data from participant registries representing real people on KRT [13], and the Global Burden of Disease (GBD) estimates for the countries, regions, or KRT modality for which the ERA Registry did not have real data (Fig. 1) (Supplementary methods) [18]. Overall, >500 000 people (511 549) are on KRT in the EU: ≈310 000 (309 905) on dialysis while ≈200 000 (201 120) are kidney graft recipients (Fig. 1). According to GBD estimates, there were ≈200 000 (205 020) people on KRT in 1990 in countries currently in the EU, so the increase has been ∼150% in 35 years [18]. Overall, for 2023, real ERA Registry KRT prevalence data for 2023 were a median of 8.1% higher than GBD estimates (range −8% to +136%) for countries in which both real ERA Registry data and GBD estimates were available. Every EU country had people on KRT. However, the KRT ranking did not closely match the population ranking (Germany 84; France 66; Italy 59; Spain 49 million). France had the highest number of people on KRT, followed by Germany, and more people were on KRT in Spain than in Italy (Fig. 2a). By contrast, the ranking for the number of people on dialysis more closely matched the population ranking for the larger countries: Germany > France > Italy > Spain (Fig. 2b). Differences in the number of kidney transplant recipients may explain in part the mismatch between overall population and people on KRT (Fig. 2c). France was the country with most kidney transplant recipients followed by Spain. In relative terms, prevalence per million population ranged from 560 (Luxembourg) to 2022 (Portugal) (Figure 3a). There was striking heterogeneity in the ratio of prevalent functioning kidney transplant to prevalent dialysis (Fig. 3b). In seven countries this ratio was >1.0, i.e. most people on KRT had a kidney graft, providing a benchmark of achievable results. This is the optimal situation. By contrast, in six countries, >75% of the KRT population was on dialysis.

**Figure 1:**
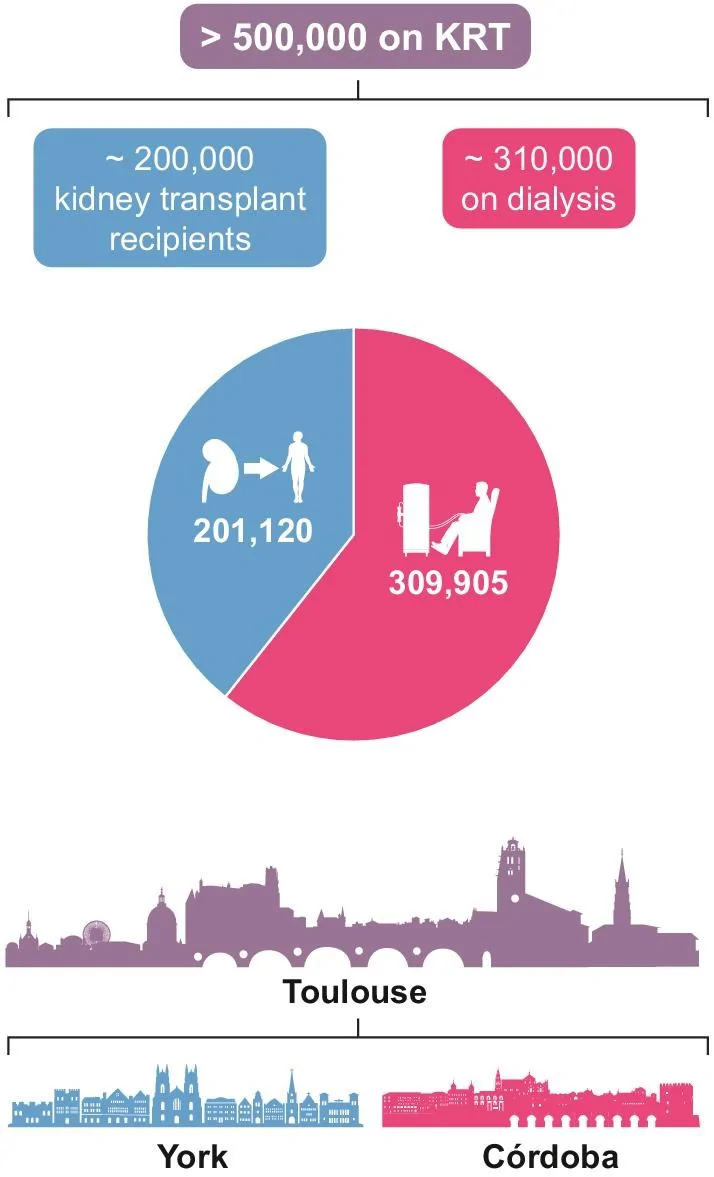
Over 500 000 people are on KRT in the EU, most of them on dialysis. Roughly, the number of people on KRT in the EU corresponds to the population of Toulouse (France), the number of kidney transplant recipients to the population of York (England), and the number of people on dialysis to the population of Cordoba (Spain).

**Figure 2:**
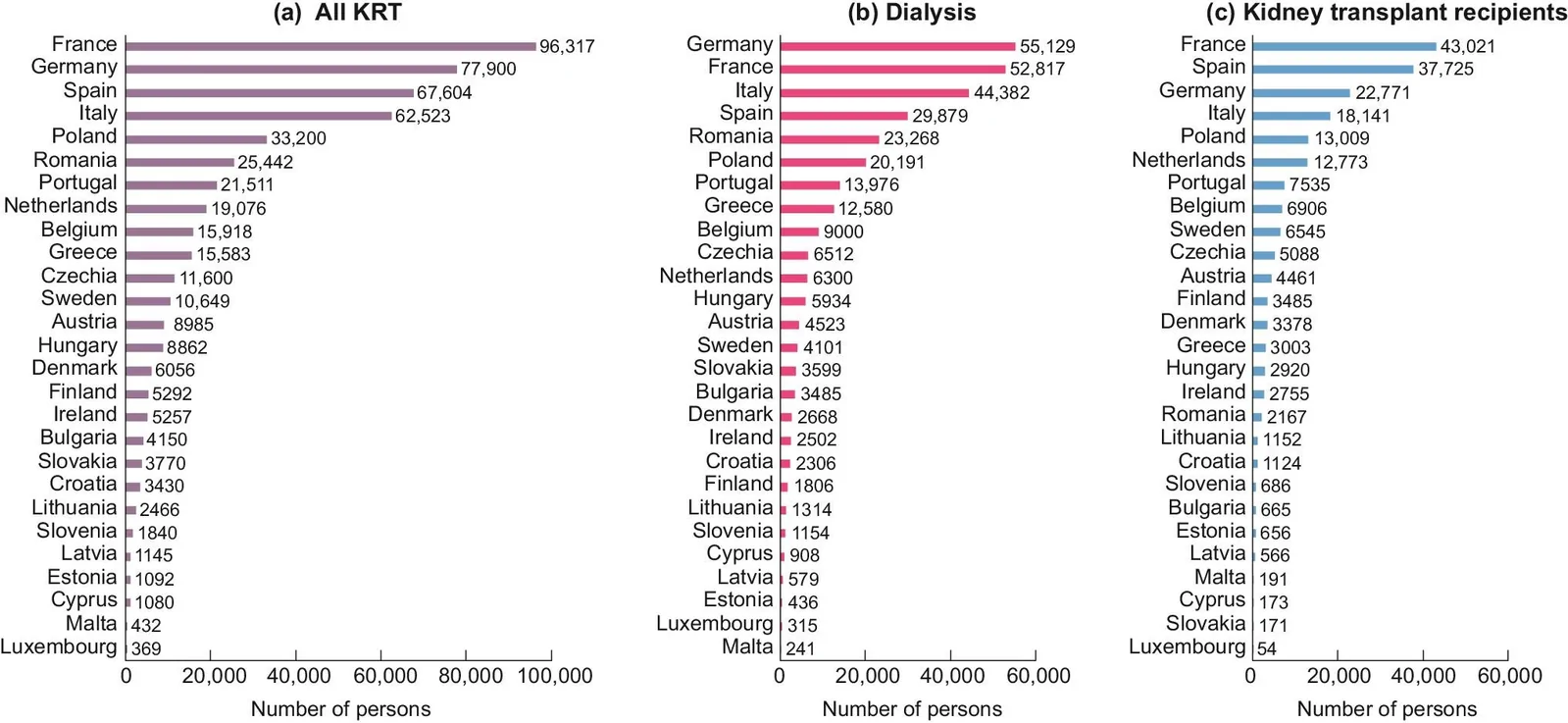
Distribution by country of people on KRT in the EU. (a) All KRT. (b) Dialysis (hemodialysis and peritoneal dialysis). (c) Kidney transplant recipients, i.e. person with a functioning kidney graft. Each panel shows the number of people on KRT or on KRT modalities (prevalence).

**Figure 3:**
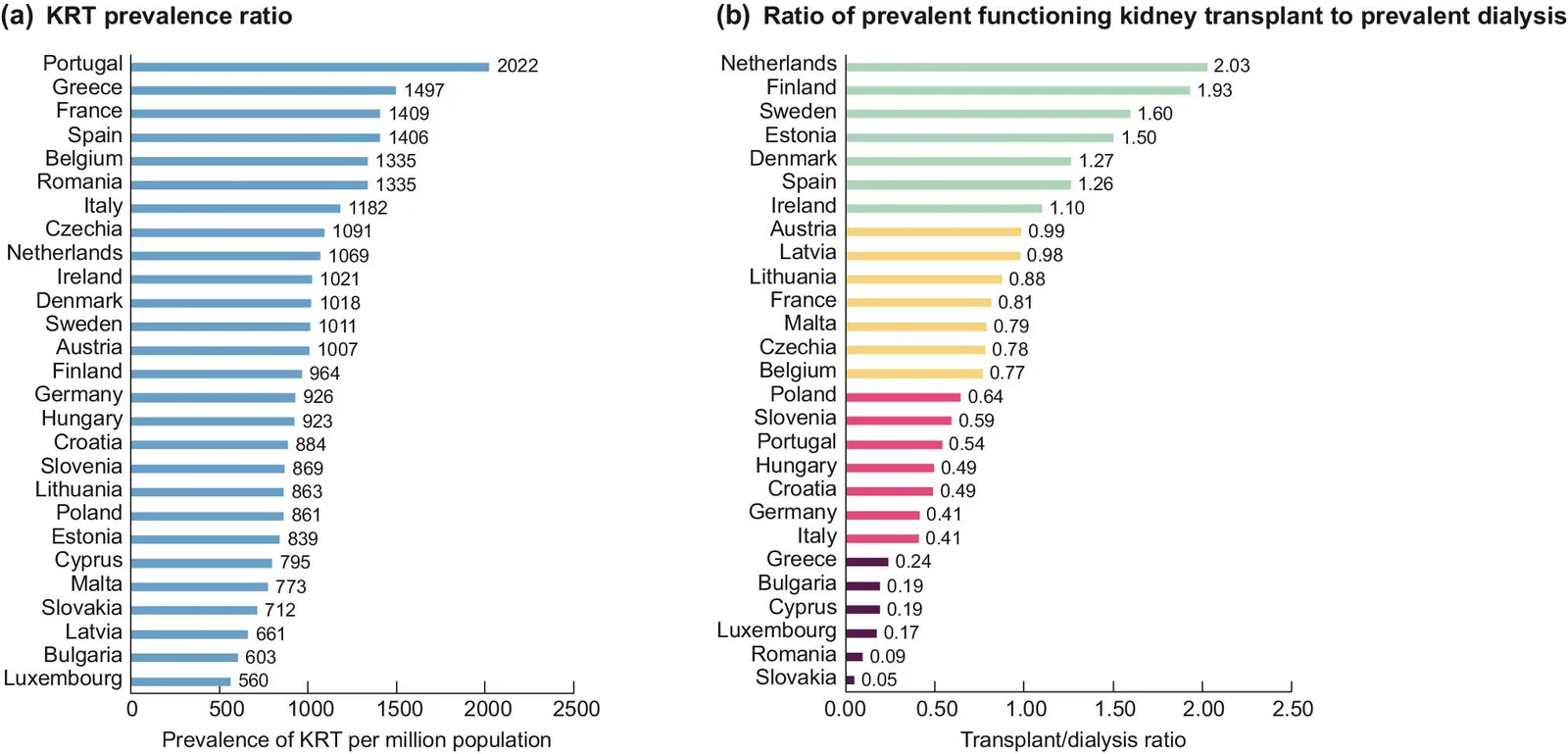
Estimated prevalence rate of KRT and transplantation/dialysis ratio. (a) Estimated prevalence of KRT per million population. Real 2023 ERA Registry data were used, unadjusted, except when data were missing, in which case, all-age prevalence data from GBD 2023 estimates were used. Note that for countries in which both datasets were available, GBD estimates underestimated KRT prevalence by a median of 85 pmp (9%), range from overestimation by 70 pmp (7%, Austria), to underestimation by 603 pmp (59%, Ireland). (b) Ratio of prevalent kidney transplantation to prevalent dialysis. The number of prevalent people with a functioning kidney graft was divided by the number of people on dialysis.

The main factors contributing to the prevalence of KRT (number of people on KRT at a given moment, usually estimated the last day of the year) are the incidence of KRT (number of people newly starting KRT) and survival on KRT (how long people live on KRT). KRT incidence and prevalence are highly heterogeneous across European countries [19]. As recently reviewed, many factors contribute to the incidence of KRT, ranging from access to KRT to the efficacy of the healthcare system in preventing and implementing early treatment for CKD [20]. In some countries access to lifesaving KRT is limited. By contrast, the main driver of survival on KRT is receiving a kidney graft as opposed to remaining in dialysis [13]. In the ERA Registry, 5-year survival was 41% on dialysis and 85% to 95% for kidney transplant recipients. In this regard, at age 20–24 years, remaining life expectancy on dialysis ranged from 36 years (men) to 44 years (women), shorter than in the general population, whereas for kidney transplant recipients it was 20 and 22 years shorter, respectively [21]. While this may be shocking, it is important that healthcare authorities are aware of these suboptimal results and ramp up CKD prevention efforts as well as screening for early CKD by testing albuminuria [22, 23]. Initiating guideline-directed treatment of CKD when albuminuria is high, yet GFR is still normal, may delay the need for dialysis for up to 27 years [24]. However shocking, the suboptimal results of KRT are not unexpected: dialysis only replaces ∼5% of the kidney function of clearing uremic retention solutes and toxins and is still marred by biocompatibility issues, as exemplified by the need for anticoagulation during hemodialysis sessions [25]. Thus, patients on dialysis remain at a functional stage of kidney failure (GFR <15 ml/min/1.73 m^2^). Furthermore, key kidney functions are not replaced by dialysis, such as their gerosuppressor function. The kidneys produce the anti-aging protein Klotho, a function lost as a consequence of albuminuria and nephron loss [26]. Thus, the CKD-associated accelerated biological aging persists while on dialysis [27]. While kidney transplantation clearly improves results over dialysis, the graft is still a single kidney, usually from a middle-aged or elderly person, which has suffered ischemia-reperfusion and at least some degree of rejection.

There were also EU-wide differences in modality of dialysis (supplementary Methods) [13, 18, 28, 29]. An overwhelming majority of EU citizens on dialysis were on hemodialysis (*n* = 286 231) (Fig. 4a), a technique that usually requires traveling to dialysis facilities thrice weekly to undergo hemodialysis for ∼4 hours each time. Around 22 700 were on peritoneal dialysis (Fig. 4b), which represents self-care home therapy. The hemodialysis/peritoneal dialysis ratio ranged from 52.0 (Slovakia) to 3.4 (Sweden) (Fig. 4c).

**Figure 4:**
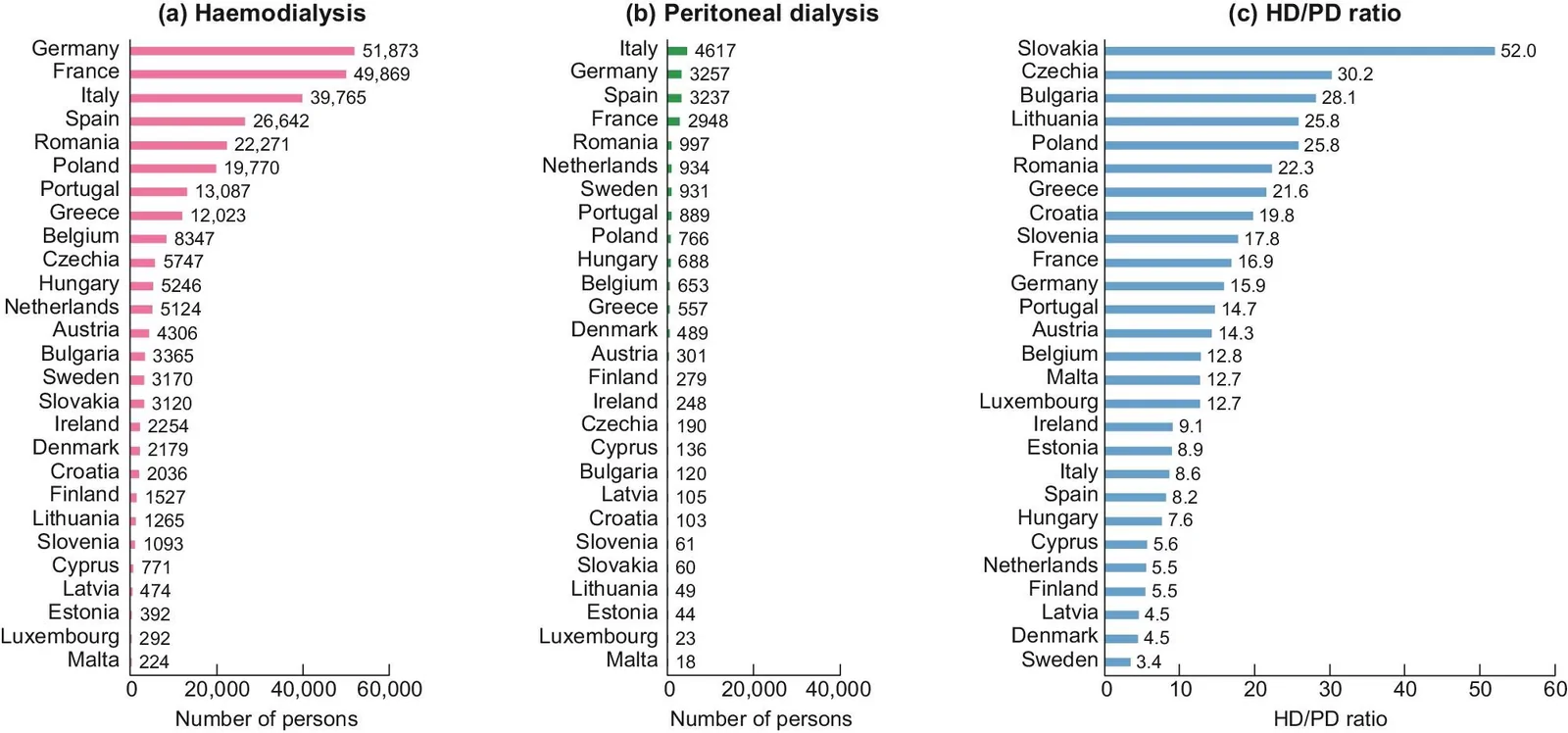
Distribution by country of people on hemodialysis and peritoneal dialysis in the EU. (a) Hemodialysis. (b) Peritoneal dialysis). (c) Ratio between the number of patients on hemodialysis and on peritoneal dialysis. (a) and (b) show the number of people on different dialysis modalities (prevalence).

The EU encompasses only part of the European countries. GBD estimates that, in countries with at least part of the territory in Europe, there were 716 700 people on KRT in 2023, most of them (484 000) in western Europe, but also in central Europe (99 300), eastern Europe including Russia (51 500), and Turkey (81 900) [18].

It is crucial for EU authorities to recognize that >500 000 citizens need KRT, most of whom are undergoing maintenance dialysis. On the one hand, this number is too high (too many patients have kidney failure) and, on the other hand, the number is too low (many patients who have kidney failure do not have access to KRT). Regulations and healthcare planning should be optimized to improve the prevention, early detection, and treatment of CKD as well as to provide a continuous improvement approach to dialysis procedures and kidney transplantation. This should be complemented by increased funding for translational, clinical and implantation research on kidney health, following the recent guidance by the World Health Organization (WHO) and the United Nations [30, 31]. This also entails that all EU countries should have a nationwide KRT registry, that enables monitoring of KRT epidemiology as a means of quality control and benchmarking. KRT registries have demonstrated the feasibility of providing kidney transplantation for a majority of KRT patients, a feature achieved in some countries. The prevalence of kidney transplantation among patients on KRT of >50% should be a quality-of-care key performance indicator of healthcare systems, since kidney transplantation offers lower costs for society, better job opportunities, quality of life and survival for patients, and a lower environmental impact.

## Supplementary Material

gfag017_Supplemental_File

## Data Availability

No new data were generated or analyzed in support of this research.
