# Supplementary material for: Over 500 000 people on kidney replacement therapy in the European Union
Source: Nephrol Dial Transplant. 2026 Feb 6;41(9):1730–5. doi: 10.1093/ndt/gfag017 (PMC13518089; doi:10.1093/ndt/gfag017)
Supplement: Supplementary file 1 [file gfag017_supplemental_file.docx]

**Supplementary methods**

The 2022 ERA Registry was considered the most reliable source for EU countries covered by the registry (1). For counties not covered by the ERA Registry, prevalence estimates were obtained from the Global Burden of Disease 2023 estimates (2). Since GBD provides data as all age prevalence per 100,000, the number of prevalent patients was calculated using GBD 2021 population data obtained from Eurostat <https://ec.europa.eu/eurostat/databrowser/view/demo_pjan__custom_18288436/default/table>, which were also used to obtain country-wide estimates for countries were only partial regional data were available (France, Italy). Hemodialysis and peritoneal dialysis estimates that could not be extracted from ERA Registry reports were obtained from the literature (3) and from the latest United States Renal Data System (USRDS) report (https://usrds-adr.niddk.nih.gov/2024/).

**References for supplemental methods**

1. Boenink R, Bonthuis M, Boerstra BA, et al. The ERA Registry Annual Report 2022: Epidemiology of Kidney Replacement Therapy in Europe, with a focus on sex comparisons. Clin Kidney J. 2024;18(2):sfae405. Published 2024 Dec 12. doi:10.1093/ckj/sfae405
2. GBD 2023 Kidney Failure with Replacement Therapy Collaborators. Global, regional, and national prevalence of kidney failure with replacement therapy and associated aetiologies, 1990-2023: a systematic analysis for the Global Burden of Disease Study 2023. Lancet Glob Health. 2025;13(8):e1378-e1395. doi:10.1016/S2214-109X(25)00198-6
3. Stel VS, de Jong RW, Kramer A, et al. Supplemented ERA-EDTA Registry data evaluated the frequency of dialysis, kidney transplantation, and comprehensive conservative management for patients with kidney failure in Europe. Kidney Int. 2021;100(1):182-195.
